# Malignant adenomyoepithelioma of the breast with axillary metastasis: a case report in a patient undergoing *EGFR*-targeted therapy and literature review

**DOI:** 10.3389/fonc.2026.1781828

**Published:** 2026-03-18

**Authors:** Qiang Zhang, Xiaoying Bai, Yaoyao Guo, Li Chen

**Affiliations:** 1 Department of Pathology, Daying People’s Hospital, Suining, Sichuan, China; 2 Department of Pathology, Deyang Hospital Affiliated Hospital of Chengdu University of Traditional Chinese Medicine, Deyang, Sichuan, China

**Keywords:** breast, clinical-pathological features, *EGFR*, lung adenocarcinoma, malignant adenomyoepithelioma

## Abstract

Malignant Adenomyoepithelioma (MAME) is a very rare tumor derived from the mixed proliferation of glandular epithelium and muscular epithelium. It has a wide spectrum of histomorphology, a high risk of local recurrence and distant metastasis, and there are few reports of axillary lymph node metastasis. This article reports a case of a breast nodule that had persisted for over 20 years and progressively enlarged during targeted therapy for pulmonary adenocarcinoma. It was eventually diagnosed as malignant adenomyoepithelioma of the breast, and axillary lymph node metastasis was later found. The sensitivity of preoperative imaging is low, and the diagnosis relies on postoperative pathology and immunohistochemistry. The efficacy of adjuvant therapy (chemotherapy or radiotherapy) is uncertain and mostly based on individual experience. Therefore, this article, in combination with previous research, comprehensively analyzes the clinical and pathological characteristics, treatment methods, and prognosis of this disease to enhance the understanding of this tumor and optimize its management strategy.

## Introduction

Breast adenomyoepithelioma (AME) is a tumor of the breast gland epithelium and myoepithelium, which was first reported by Hamperl (1) in 1970. Malignant adenomyoepithelioma (MAME) is a rare condition, with the majority of reported cases worldwide being individual instances. However, there have been no reported cases of MAME progression with axillary lymph node metastasis following targeted therapy for lung adenocarcinoma. This unique confluence raises intriguing questions about potential shared pathogenic pathways or the impact of targeted therapy on secondary tumor progression.

## Case information

**Figure 1:**
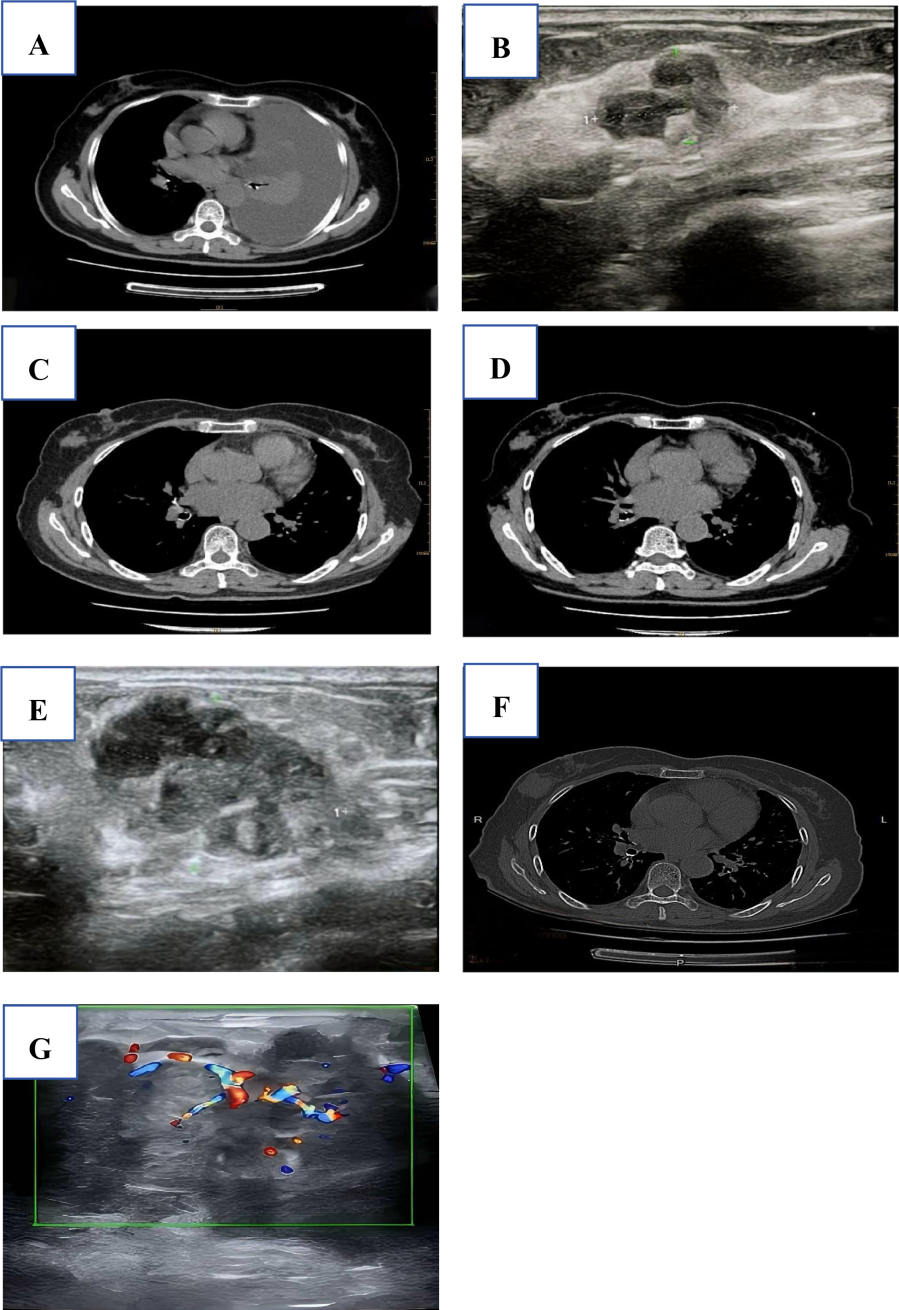
**(A)** 2019-07-02, CT scan: A well - defined nodule measuring 13.9 mm × 10.1 mm was identified in the upper outer quadrant of the right breast. **(B)** 2019-10-15, Breast Ultrasound: A hypoechoic nodule measuring 14.1 mm × 10.2 mm was detected in the right breast, classified as BI - RADS category 4a. **(C)** 2020-10-09, CT scan: A 21 mm ×18 mm nodular opacity was observed in the upper outer quadrant of the right breast; its etiology remains indeterminate and requires further characterization. **(D)** 2021-03-31, CT scan: A 23.3 mm × 18.6 mm nodular opacity was present in the upper outer quadrant of the right breast, exhibiting irregular morphology, relatively well - defined margins, and mild post-contrast enhancement; no significant interval change is observed compared to the prior study. **(E)** 2022-02-08, Breast Ultrasound: A 27.2 mm × 18.7 mm slightly hypoechoic mass was observed in the right breast, classified as BI - RADS category 4a. **(F)** 2022-11-08, CT scan: A 30.3 mm × 29.0 mm nodular opacity was present in the upper outer quadrant of the right breast. **(G)** 2024-04-02, Breast Ultrasound: A 59 mm × 36 mm × 42 mm slightly hypoechoic mass was observed in the breast, exhibiting irregular morphology, poorly defined margins, and heterogeneous internal echotexture.

A 74-year-old woman had a right breast nodule for over 20 years. The nodule progressively enlarged during her 5-year targeted therapy for lung cancer. In July 2019, the patient presented with “dull pain in the right chest and abdomen, palpitations, fatigue, and shortness of breath for over a month”. The chest CT scan revealed lung cancer in the left lower lobe and a nodular shadow in the upper right quadrant of the right breast with an indeterminate nature. In October 2019, the patient underwent a lung biopsy, which confirmed the diagnosis of adenocarcinoma with epidermal growth factor receptor (*EGFR*)mutations in exon 19 p.745_750del and exon 21 p.L858R. During the same period, the B-mode ultrasound revealed a hypoechoic nodule in the lateral aspect of the right breast, measuring approximately 14.1 mm × 10.2 mm, BI-RADS 4a. The patient was diagnosed with stage IV lung cancer. After receiving treatment with oral gefitinib and undergoing regular follow - up examinations, tumor progression was observed. This prompted the initiation of osimertinib as a targeted therapeutic intervention. Progressive enlargement of breast nodules has been identified through multiple CT or ultrasound examinations (**Figure 1**). In April 2024, a breast ultrasound examination revealed a hypoechoic mass in the right breast at the 9–10 o’clock position, measuring approximately 59mm×36mm×42mm. The mass had an irregular shape, indistinct margins, and heterogeneous internal echogenicity. No enlarged lymph nodes were detected in the axillary region. The findings indicate the presence of a hypoechoic mass in the right breast, BI-RADS 4c. Two months later, after excluding surgical contraindications, a biopsy was performed. The pathological result indicated an epithelial - muscle tumor, which was consistent with atypical adenomyolipoma. Given the large size of the mass and the limited biopsy tissue, surgical treatment was recommended, along with an intraoperative frozen section to further rule out the possibility of more severe lesions. Intraoperative frozen section pathology diagnosis revealed an epithelial-myoepithelial tumor with necrosis, indicating a potential malignant adenomyoepithelioma. In this situation, the surgeon communicated with the patient’s family. The family refused to have the patient undergo a modified radical mastectomy for breast cancer, so only a tumor excision was performed. Finally, the pathological diagnosis confirmed a malignant adenomyoepithelioma with necrosis in the right breast.

**Figure 2:**
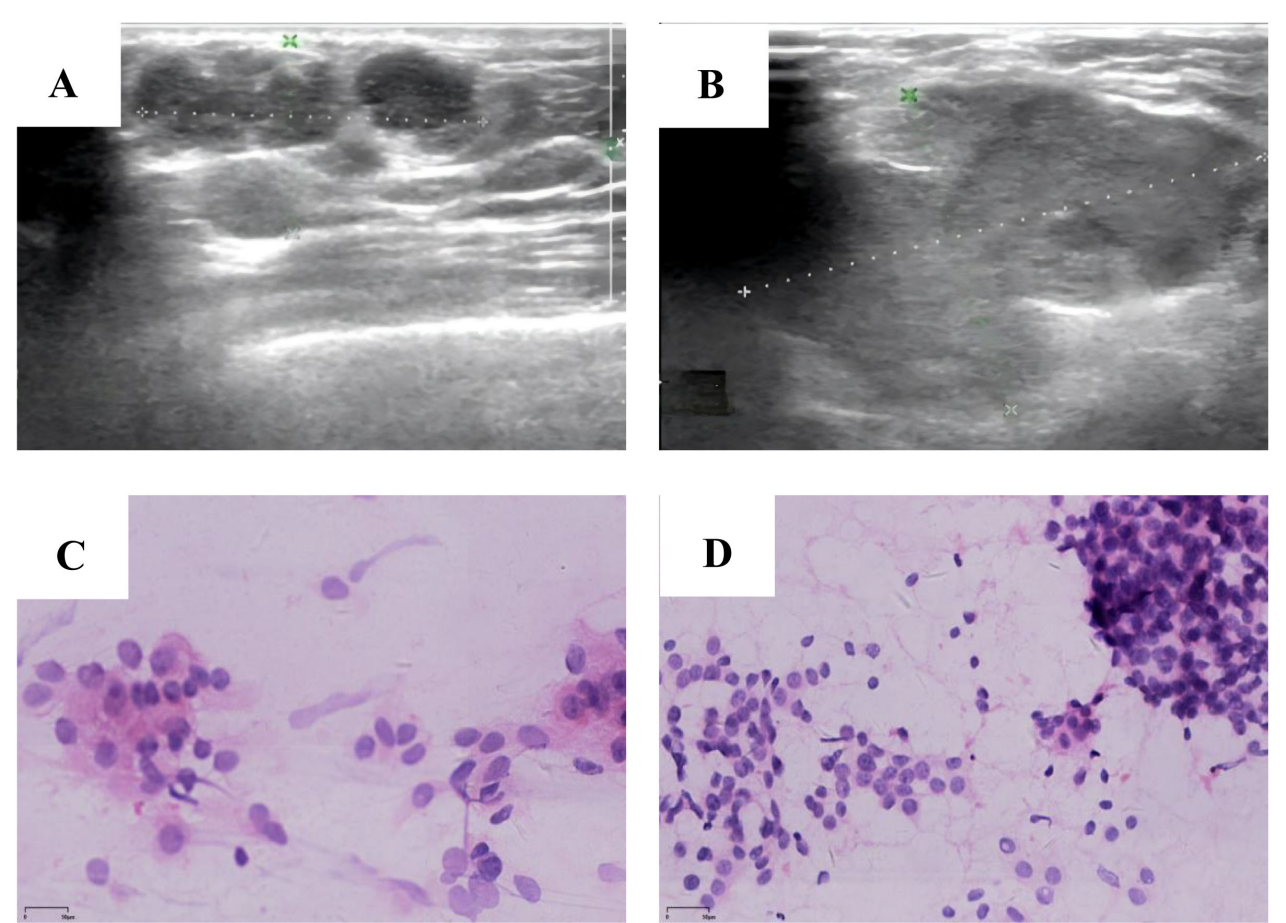
**(A)** 2025-01-04, A hypoechoic nodule measuring 28.0 mm × 17.7 mm was detected in the right breast's upper outer quadrant, classified as BI-RADS 4c. **(B)** 2025-01-04, Enlarged lymph nodes were detected in the right axilla, measuring 45.5 mm × 33.1 mm. **(C)** A tumor cell mass was identified in the cytological examination of the right breast aspirate. (HE×200) **(D)** A tumor cell mass was identified in the cytological examination of the right axillary lymph node aspirate. (HE×200).

**Figure 3:**
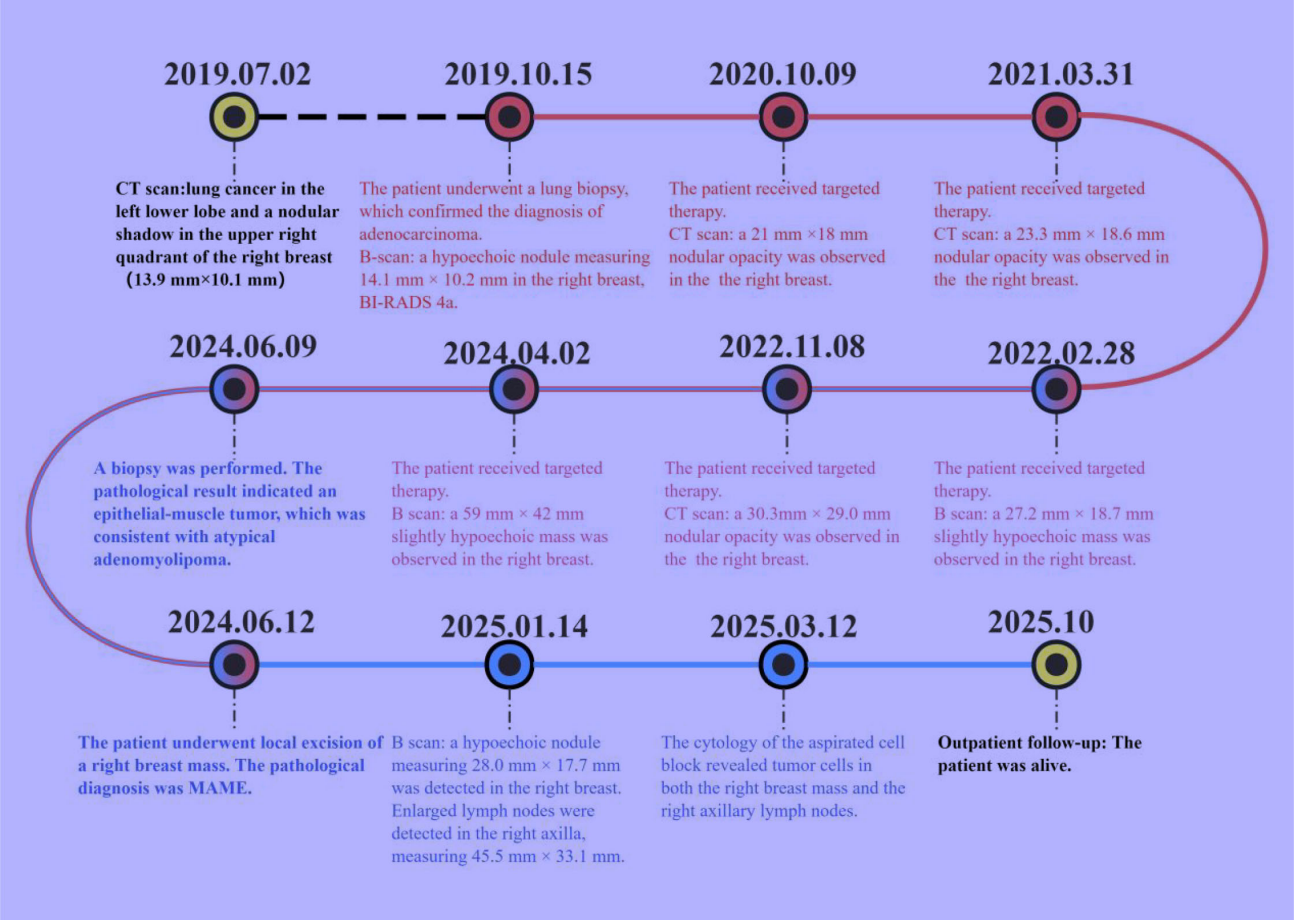
Timeline of patient consultations.

On January 4, 2025, B-mode ultrasound revealed multiple isoechoic nodules in the upper outer quadrant of the right breast, with the largest measuring 19.1 × 14.0 mm. These lesions demonstrated ill-defined margins, lack of a discrete capsule, irregular morphology, and partial coalescence, forming a confluent tumor mass approximately 28.0 mm × 17.7 mm in size (**Figure 2A**). The internal echotexture was heterogeneous, and minimal vascularity was observed on Doppler imaging. No additional significant abnormalities were detected within the remaining breast parenchyma. Notably, an irregular isoechoic nodule measuring 45.5 × 33.1 mm was identified at the right axillary apex, exhibiting poorly defined borders, heterogeneous internal echoes, and scant vascular signals (**Figure 2B**). A hypoechoic nodule is identified in the right breast, classified as BI-RADS category 4c, accompanied by suspicious regional lymphadenopathy in the ipsilateral axilla. Two months later, fine-needle aspiration was performed on both the breast nodule and the ipsilateral axillary lymph node. Cytological and cell block examinations confirmed tumor recurrence with metastasis to the ipsilateral axillary lymph node. The patient’s entire visit timeline is shown in **Figure 3**.

## Pathological examination

### Gross examination

**Figure 4:**
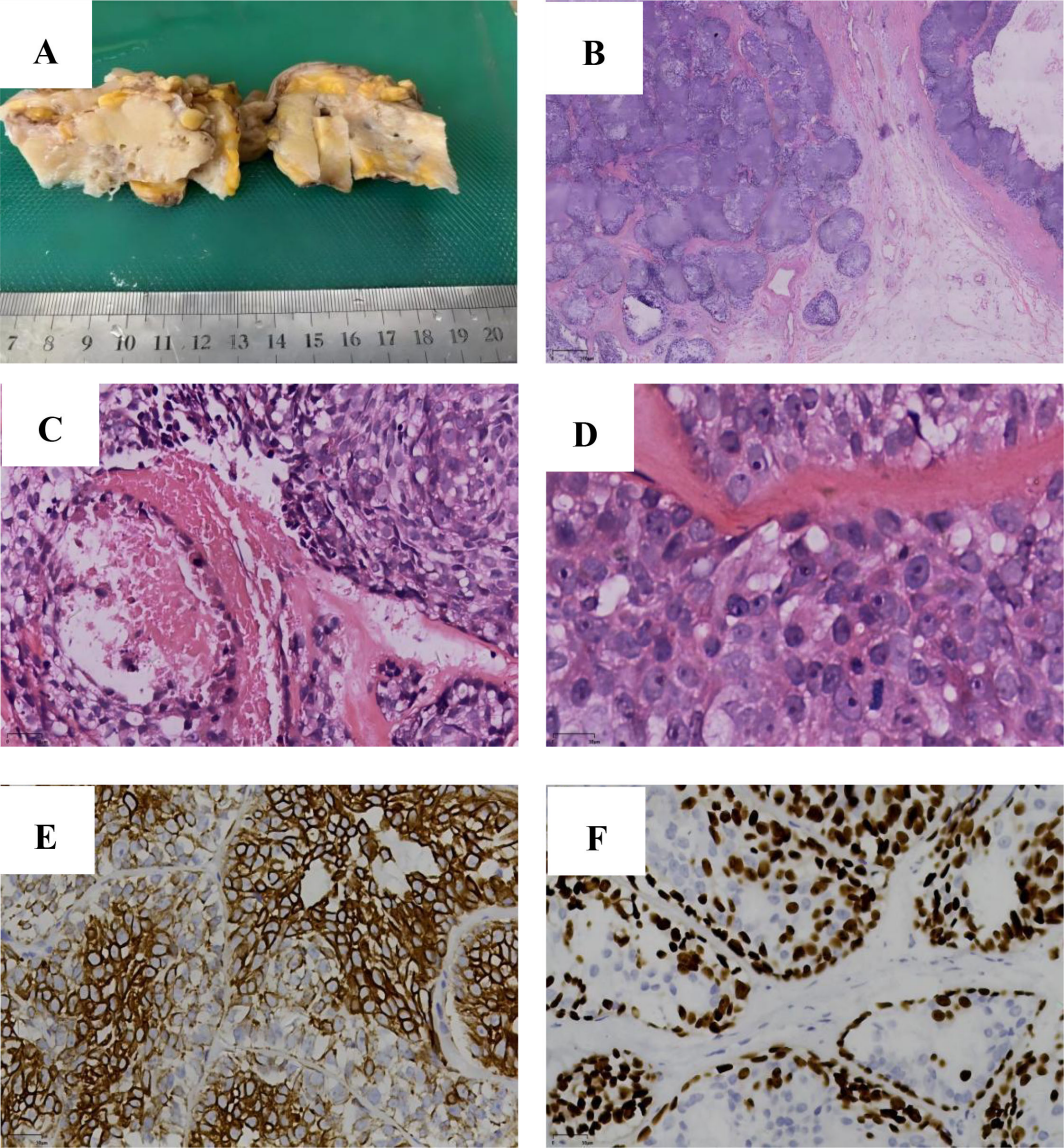
**(A)** Gross Pathological Examination: The tumor specimen presents a grayish - white to grayish - yellow cut surface, with cystic and solid areas. It has a moderate consistency and well - demarcated margins. **(B)** Histopathology (Low Power): The tumor demonstrates expansile growth with focal infiltration into the adjacent parenchyma. **(C)** Two morphologically distinct tumor cell populations display a biphasic tubular arrangement, and central necrotic debris is observed. (HE×200). **(D)** Glandular epithelial cells possess eosinophilic cytoplasm, while myoepithelial cells have clear cytoplasm. Pathological mitoses are present. (HE×400). **(E)** CK8 shows strong positivity in the glandular epithelium and weak positivity in some myoepithelium. (EnVision×200). **(F)** SOX10 expression is positive in myoepithelial cells. (EnVision×200).

A skin-bearing tissue specimen, measuring 8 cm × 4 cm × 4 cm, was observed. A subcutaneous mass, measuring 6 cm × 4 cm × 4 cm and located 1 cm beneath the skin surface, was identified. Its cut surface showed grayish - white, grayish - yellow, and yellowish hues. The mass demonstrated a cystic-solid consistency, was slightly firm to palpation, and exhibited mucinous degeneration (**Figure 4A**). The demarcation between the mass and adjacent tissues was well defined. Microscopic findings: The tumor exhibits expansile-infiltrative growth pattern with a predominant nested architecture. Two distinct cell populations are arranged in a double-layered tubular configuration, surrounded by an eosinophilic basement membrane. Central coagulative necrosis is observed within the cellular nests. Focal areas show glandular differentiation, with lumens containing eosinophilic, homogeneous secretions (**Figures 4B, C**). High-power microscopic examination reveals a biphasic tumor composed of glandular epithelial and myoepithelial (**Figure 4A**) cells. Glandular epithelial cells exhibit well-defined cell borders and are round to oval in shape, with eosinophilic or foamy cytoplasm. The nuclei are markedly enlarged, display marked nuclear atypia, possess distinct nuclear membranes, contain finely granular chromatin, and harbor prominent nucleoli.

Myoepithelial cells have poorly defined cell margins and clear cytoplasm. Their nuclei show predominantly moderate atypia with focal areas of severe atypia; nuclear membranes are clearly delineated, chromatin is fine and evenly distributed, and nucleoli are evident in some nuclei. Pathological mitotic figures are present in both cellular components (**Figure 4D**). Immunophenotype: The tumor exhibits a biphasic immunoprofile. Glandular epithelial cells are positive for GATA-3 and CK8 (strong membranous and cytoplasmic staining, **Figure 4E**), with membranous expression of β-catenin and E-cadherin. CK5/6 shows focal positivity, and estrogen receptor (ER) is moderately expressed in approximately 60% of tumor nuclei. Myoepithelial cells express SOX-10 (nuclear staining, **Figure 4F**), smooth muscle actin (SMA), p63, and show partial CK5/6 positivity; glial fibrillary acidic protein (GFAP) and ER are negative. Both cellular components demonstrate heterogeneous nuclear expression of c-MYC, while the progesterone receptor (PR) is negative, human epidermal growth factor receptor 2 (Her - 2) immunoreactivity is absent (score 0). The p53 staining pattern is consistent with wild-type expression. Ki67 labeling index reaches up to 50% in proliferative hotspots. Mismatch repair proteins (MLH1, PMS2, MSH2, and MSH6) are retained in both components. Pathological Diagnosis: Malignant adenomyoepithelioma of the right breast, exhibiting extensive tumor necrosis.

Cytological and cell block examinations confirmed tumor recurrence with metastasis to the ipsilateral axillary lymph node (**Figures 2C, D**). Immunohistochemical analysis of the paraffin-embedded tissue from the right breast mass biopsy revealed that tumor cells were positive for GATA-3, negative for ER and PR, and exhibited Her-2 expression at a 1+ intensity in approximately 15% of tumor cells. CK5/6 and p63 showed focal positive immunoreactivity, and the Ki67 proliferation index was approximately 40%. In the lymph node biopsy specimen, tumor cells displayed focal positivity for GATA-3, were negative for ER and PR, and showed no evidence of Her-2 overexpression. CK5/6 and p63 demonstrated diffuse positive immunoreactivity, with a Ki67 proliferation index of approximately 25%.

## Discussion

According to the fifth edition of the WHO classification of breast tumors, MAME is categorized into two subtypes: AME with carcinoma and epithelial-myoepithelial carcinoma (2). In 2021, Rakha et al. (3) classified AME into benign, atypical, and MAME, with MAME further subdivided into *in situ*, invasive, and invasive-associated AME. The diagnostic features suggestive of malignancy include an infiltrative border, marked cellular atypia, increased mitotic activity (>3/10HPF), tumor necrosis, and the presence of satellite nodules. When only a subset of these malignant characteristics is observed, the lesion is classified as atypical AME. In this case, the breast biopsy specimen lacked clear margins and yielded a limited pathological diagnosis of atypical AME due to tissue constraints. Although the frozen section analysis from the local excision provided additional information on tumor margins, a single slide could not fully reflect the tumor’s overall characteristics, suggesting MAME. Following adequate sampling of the frozen-to-paraffin tissue and with the assistance of immunohistochemistry, the final diagnosis was confirmed as MAME.

Histologically, the fifth edition of the WHO Classification of Breast Tumors (2) indicates that MAME displays a wide range of morphological features. When the malignant component involves glandular epithelium, the tumor may exhibit histological patterns resembling invasive carcinoma of no special type, invasive lobular carcinoma, or other special types of breast carcinoma; therefore, extensive sampling is essential to identify representative diagnostic areas. In cases where malignant transformation occurs in the myoepithelial component, the lesion is characterized by atypical myoepithelial cells with clear or eosinophilic cytoplasm and spindle-shaped, epithelioid, or plasmacytoid morphology, along with marked nuclear atypia and increased mitotic activity (>3/HPF). Rarely, both epithelial and myoepithelial components may undergo concurrent malignant transformation, resulting in a biphasic neoplasm composed of intermixed malignant epithelial and myoepithelial cells. The immunophenotype showed strong positivity for CK8/18, EMA, and CK7 in the glandular epithelial component, and diffuse or focal positivity for S100, SMA, p63, calponin, and smooth muscle myosin heavy chain (SMMHC) in the myoepithelial component.

The breast tumor in this case had a clinical history of over 20 years, and progressive enlargement was observed in the past 5 years. We hypothesize that the MAME in this study evolved from a pre-existing benign AME, which is consistent with the established concept that MAME arises from benign adenomatous lesions-potentially originating from pleomorphic adenoma or benign myoepithelioma and may be associated with myoepithelial cell hyperplasia (4, 5). Studies have demonstrated that the p53 protein is expressed in the nuclei of both AME and MAME, suggesting the presence of TP53 mutations and their potential role as early events in tumorigenesis (6). In this case, however, p53 immunoreactivity showed a wild-type pattern. In MAME, ER, PR, and HER2 are typically negative, with molecular alterations predominantly involving mutations in *PIK3CA* and *HRAS p.Gln6*1 (7). Notably, *HRAS p.Gln61* mutations are associated with atypical histological features of ER - negative MAME, whereas *PIK3CA* mutations are more commonly observed in ER - positive malignant adenomyoepithelioma. Homozygous deletion of *CDKN2A* may contribute to the malignant transformation of ER-negative AME. *HRAS* mutations at codons G12D, G13R, and G12S have been reported in a small number of cases. Notably, ER and PR negative MAME may harbor concurrent *PIK3CA H1047R* and *HRAS* G12/G13 hotspot mutations; *EGFR* mutations have additionally been detected in isolated case reports (8). Furthermore, MAME has been reported to coexist with Lynch syndrome. In this case, no loss of expression was observed for the mismatch repair proteins (MLH1, PMS2, MSH2, and MSH6) associated with Lynch syndrome (9). Genetic testing revealed an *EGFR* exon 19 deletion (p.E746 - A750del) and an *EGFR* exon 21 mutation (p.L858R) in the patient’s lung adenocarcinoma. The patient had received targeted therapy with *EGFR* tyrosine kinase inhibitors (*EGFR-TKIs*) -gefitinib or osimertinib - for over five years. During this period, the breast mass progressively increased in size. Whether the malignant progression of MAME is associated with long - term targeted therapy remains to be elucidated. Current reports indicate that adverse effects of *EGFR-TKIs* include interstitial lung disease (10). Individual case studies (11) have suggested a potential temporal association between long - term use of *EGFR-TKIs* and the development of multiple myeloma. However, these observations are limited to isolated cases, and no definitive causal relationship has been established. Studies suggest that *EGFR-TKIs* may alter the tumor immune microenvironment (12) or activate compensatory signaling pathways, such as *MET*, *HER2*, and the *PI3K/AKT/mTOR* pathway (13). Therefore, we hypothesize that the observed cases in this study may be associated with the activation of the *PI3K/AKT/mTOR* compensatory signaling pathway. The mechanism underlying its occurrence and development requires further investigation.

A systematic literature retrieval was conducted in PubMed using keywords “breast,” “malignant,” and “adenomyoepithelioma.” The search was conducted on October 23, 2025. Initial screening identified 58 studies; full-text assessment excluded non-detailed studies, resulting in 45 studies with 72 patients (6, 15–56). All patients were female (34–86 years, mean 56.57 years); 65.28% (47/72) were aged over 50 years. Tumor size ranged 0.4–9 cm: <2 cm (38.89%, 28/72), 2–5 cm (41.67%, 30/72), >5 cm (9.72%, 7/72); 9.72% (7/72) had no size recorded. Axillary lymph node metastasis occurred in 8.33% (6/72) of patients. Follow-up duration: <1 year (26.37%, 19/72), 1–3 years (44.44%, 32/72), >3 years (26.39%, 19/72). MAME patients received surgery, radiotherapy, chemotherapy, or endocrine therapy. Follow-up period 0–162 months: local recurrence (9.72%, 7/72), distant metastasis (22.22%, 16/72; lung 62.5%, brain/kidney 18.75% each), mortality (22.22%, 16/72). (**Table 1**).

**Table 1:** Summary of data from 72 patients.

Characteristics	All patients n=72
Age (year)	
≦50	25 (34.72)
> 50	47 (65.28)
Size	
< 2 cm	28 (38.89)
2–5 cm	30 (41.67%)
> 5 cm	7 (9.72%)
Not recorded	7 (9.72%)
Axillary lymph node metastasis	
Yes	6 (8.33%)
No	66 (91.67%)
Follow-up time (month)	
< 12	19 (26.39%)
12-36	32 (44.44%)
> 36	19 (26.39%)
Not recorded	2 (0.37%)
Surgery	
Lumpectomy	22 (30.56%)
Lumpectomy + SLNB	8 (11.11%)
Breast-conserving surgery + SLNB	4 (5.56%)
Mastectomy	13 (18.06%)
Mastectomy and SLNB	18 (25.00%)
Modified radical mastectomy	2 (2.78%)
Radical mastectomy	5 (6.94%)
Radiation therapy	
Yes	17 (23.61%)
No	55 (76.39%)
Chemotherapy	
Yes	13 (18.06%)
No	59 (76.39%)
Hormonal therapy	
Yes	7 (9.72%)
No	65 (90.282%)
Recurrence	
Yes	8 (11.11%)
No	64 (88.89%)
Survival status	
Died	10 (13.89%)
Alive	62 (86.11%)
Distant metastasis	
Yes	18 (25.00%)
No	54 (75.00%)

After excluding 72 cases with missing tumor size/survival data, 64 cases were analyzed; adding the present case gave 65 total. Univariable (**Table 2**, **3**) and multivariate (**Table 4**) analyses showed distant metastasis and tumor size (>5 cm) as independent mortality predictors; age, lymph node metastasis, and surgical approach had no significant prognostic value.

**Table 2:** Univariate analysis for mortality.

Factor	Category	Deaths /Total	Mortality rate (%)	HR	95%CI	P-value
Age	≤50 years	4/26	15.4	1.00	–	0.098
	>50 years	11/39	28.2	2.11	0.66-6.74	
Tumor Size	<2cm	2/24	8.3	1.00	–	0.003
	2-5cm	7/32	21.9	2.91	0.60-14.09	
	>5cm	6/9	66.7	12.6	2.55-62.27	
Lymph Node Metastasis	No	13/59	22.0	1.00	–	0.324
	Yes	2/6	33.3	1.78	0.39-8.11	
Distant Metastasis	No	6/51	11.8	1.00	–	<0.001
	Yes	9/14	64.3	8.25	2.94-23.14	

**Table 3:** Univariate analysis for recurrence risk.

Factor	Recurrence /Total	Mortality rate (%)	HR	95%CI	*P* value
Age	3/26	11.5	7.7	–	0.678
	3/39	7.7	8.3	0.12-3.38	
Tumor Size	2/24	8.3	6.3	–	0.534
	2/32	6.3	22.2	0.10-5.54	
	2/9	22.2	3.9	0.38-25.78	
Distant Metastasis	2/51	3.9	28.6	–	0.028
	4/14	28.6	9.71	1.51-62.47	

**Table 4:** Multivariate analysis for mortality risk.

Variable	Adjusted HR	95%CI	P-value
Age(>50 years)	6.84	2.31-20.26	<0.001
Tumor size(>5cm)	5.92	1.35-25.98	0.018
Distant Metastasis	1.74	0.53-5.71	0.360

No universally accepted clinical guideline exists for MAME management. Complete surgical excision with histologically confirmed negative margins reduces local recurrence risk (9). MAME metastasizes hematogenously, predominantly to lung and brain (14). Routine axillary lymph node dissection role is uncertain due to rare axillary metastasis. Wang et al. (15) reported SLNB may improve MAME prognosis. Chemotherapy shows limited efficacy; targeted therapy has potential benefits (58). Our systematic review (65 studies) found no significant association between prognosis and mastectomy or lymph node dissection after complete primary tumor resection.

## Conclusions

This study reports a primary breastMAMEcase with EGFR mutation and axillary metastasis, detailing clinical manifestations, imaging features, and diagnostic/therapeutic strategies. Relevant MAME literature was reviewed.

This study summarizes clinicopathological characteristics, treatments, and prognosis of MAME patients. Distant metastasis and tumor size >5 cm were independent adverse prognostic predictors; age, lymph node metastasis, and surgical approach showed no significant prognostic association.

Key clinical recommendations for MAME management are proposed as follows:

Diagnosis: Suspected MAME requires preoperative clinical breast examination by specialists. Progressively enlarging lesions need assessment and immediate core needle biopsy for definitive diagnosis.

Surgical Management: Complete resection with histologically confirmed negative margins is standard. Axillary lymph node evaluation is essential: SLNB for detected lymphadenopathy; ALND for SLNB-positive metastasis. Mastectomy is considered for large tumors.

Adjuvant Therapy: Radiotherapy/chemotherapy role is uncertain for recurrence or metastasis. Eligible patients may receive adjuvant systemic therapies (chemotherapy, targeted therapy, endocrine therapy) after informed consent.

Follow-up Surveillance: Regular follow-up is recommended for local excision patients, especially those with risk factors (local recurrence, lymph node involvement, distant metastasis). Surveillance includes clinical evaluation and imaging for early progression detection.

## Data Availability

The original contributions presented in the study are included in the article/supplementary material. Further inquiries can be directed to the corresponding author.
